# Postoperative Outcomes and Complications in Menopaused Patients after Reduction Mammoplasty

**DOI:** 10.1016/j.jpra.2024.12.004

**Published:** 2024-12-17

**Authors:** Edward T.C. Dong, Jérôme Martineau, Gauthier Zinner, Daniel F. Kalbermatten, Carlo M. Oranges

**Affiliations:** Department of Plastic, Reconstructive, and Aesthetic Surgery, Geneva University Hospitals, Geneva University, Geneva, Switzerland

**Keywords:** Breast reduction, Menopause, Menopausal status, Outcomes

## Abstract

**Background:**

Symptomatic macromastia is a debilitating condition that affects millions of women worldwide. Although reduction mammoplasty is the gold standard treatment, the association between estrogen levels and wound healing has been established in literature. Hence, this study aimed to compare the postoperative outcomes and complications between menopaused and non-menopaused women after reduction mammoplasty.

**Method:**

This study offers a retrospective multimodal observation and analysis comparing menopaused and non-menopaused women. Using data collected from January 2018 to May 2024, patients who met the selection criteria were divided into 2 groups. Complications following reduction mammoplasty were recorded and analyzed.

**Results:**

A total of 110 patients were included in this study, among them 80 patients were in the non-menopaused group and 30 in the menopaused group. Our statistical analysis indicated that the hospital stay was significantly longer in the menopaused group (P=0.008). Additionally, postoperative dog ears were significantly more frequent in the menopaused group (P=0.034). Conversely, scar hypertrophy occurred more frequently in non-menopaused patients (P=0.02).

**Conclusion:**

Although menopaused women undergoing single or bilateral reduction mammoplasty had longer duration of hospital stay, they did not have higher risk of postoperative complications, except for higher rate of developing dog ears, which may be ascribed to the faltering estrogen levels of this population. Non-menopaused women had a higher rate of hypertrophic scars.

## Introduction

Mammary hypertrophy, otherwise known as gigantomastia or macromastia is a debilitating medical condition that affects millions of women worldwide.^1^^,^^2^ In most instances, the rapid and massive growth of breast tissue is either instigated by puberty, and is thus referred to as virginal breast hypertrophy, or can be triggered by gestation, and referred to as gravid breast hypertrophy.^3^, ^4^, ^5^, ^6^, ^7^ Hypertrophic breasts can cause a wide range of physical suffering including chronic neck, shoulder, and back pain, kyphosis, neuropathies, and persistent intertriginous rashes in the infra-mammary fold (IMF).^2^, ^3^, ^4^, ^5^ These symptoms are frequently accompanied by physical discomfort such as difficulties in dressing, sleeping, breathing, and exercising.^3^^,^^6^

Moreover, patients affected by symptomatic breast hypertrophy also experience psychological distress, often considering themselves socially inadequate, with their breast size interfering in their sexual life and self-esteem.^3^^,^^6^^,^^7^ Conservative treatments such as weight loss, physical therapy, and medication have been proven to lack in efficacy.^8^^,^^9^ Consequently, surgery, particularly, reduction mammoplasty (RM) has become the gold standard treatment.^2^ With a high satisfaction rate, RM is the tenth most performed surgical procedure by plastic surgeons according to the International Society of Aesthetic Plastic Surgery (ISAPS) in their 2023 international survey.^10^ Free nipple grafting, suction lipectomy, and numerous pedicle designs can be selected to perform this operation. Although the inferior pedicle, first introduced by Ribeiro in 1975 with the Wise-pattern incision being the most common approach in the United States,^11^, ^12^, ^13^ the superomedial, superior, and vertical pedicles have proven to be reliable options as well.^14^, ^15^, ^16^, ^17^

Nonetheless, this surgery can be associated with serious postoperative complications, including hematomas, seromas, infections, and nipple-areola complex (NAC) necrosis.^2^^,^^18^

The current literature has established associations between estrogen levels and wound healing,^19^ but the exact mechanism linking the 2 is yet to be determined.^20^ Although estrogen deficiency and unfavorable wound healing caused by physiological and architectural changes appear to be correlated,^5^^,^^21^^,^^22^ there is a paucity of literature evaluating the effects of menopause on surgical outcomes, especially in breast reduction. Herein, we present our observations and analyses on the outcomes and complications of RM in the advent of menopause.

## Methods

We performed a retrospective chart review of all female patients who underwent RM from January 2018 to May 2024. All cases of oncoplastic mammary reduction were excluded.

Patients’ age, smoking status, body mass index (BMI), comorbidities, and previous medical interventions were collected from anesthesiologic and medical charts. Operative reports were reviewed for surgical details such as the pedicle type and resection weight. The menopausal status was either confirmed from the medical charts or via discussion with the patients. We screened hospital letters and outpatients reports to assess the duration of hospital stay and postoperative complications. Complications were defined as early if they occurred less than 30 days post-surgery and as late if they occurred more than 30 days post-surgery.

The approval by the competent institutional review board (project ID: CCER 2024-01071) was obtained. Informed consent was obtained from all the included patients.

### Preoperative measures

A single metric tape was used to perform preoperative measurements. This was performed by the plastic surgeon or a plastic surgery fellow. The measurements included the NAC diameters, sternal notch-to-nipple distance (SN-N), and inferior border of NAC to IMF distance. Breast asymmetry was also preoperatively recorded.

### Statistical analysis

Patient characteristics were incorporated in an Excel spreadsheet (Version 16.83, Microsoft Corp., Redmond, WA, USA). Using GraphPad Prism version 10.3.0, the Shapiro-Wilk test was performed to verify the assumption of normality. We compared the patients using two-tailed *t*-test and Mann-Whitney test. The two-sided Chi-squared or Fischer's exact tests were used when analyzing categorical variables. A linear regression was performed to assess confounders. The statistical significance was set at P value < 0.05.

## Results

### Patient selection

Overall, 354 RM procedures were performed in the selected period. After retrospective analysis and application of the inclusion and exclusion criteria, 190 successive interventions were retained. Informed consent was not provided by 80 patients and they were therefore excluded, leading us to the inclusion of 110 women, representing 80 women in the non-menopaused (63.3%), and 30 women in the menopaused (36.7%) groups (Table 1).

**Table 1:** Baseline characteristics

	Non-menopaused	Menopaused	
	*n=80*	*n=30*	*P-value*
Age [years], mean (SD)	32.7 (12.64)	57.3 (6.51)	<0.001*
BMI [kg/m^2^], mean (SD)	25.6 (2.98)	27.6 (4.01)	0.005
Active smokers, n (%)	16.0 (20%)	5.0 (16.7%)	0.692
**Comorbidities**			
Diabetes, n (%)	2 (2.5%)	3 (10%)	0.093
Hypertension, n (%)	4 (5%)	4 (13.3%)	0.13
Coagulopathy, n (%)	3 (3.75%)	0	0.019*
**Follow-up [months], mean (SD)**	9.4 (8.10)	10.2 (10.70)	0.89

### Preoperative assessment measurements

The average age of menopaused patients was 57.3 years (SD 6.5 years) compared to 32.7 years (SD 12.6 years) in non-menopaused patients (P<0.001). Except for the menopausal status and age, the 2 groups were similar in terms of baseline characteristics, preoperative measurements, and surgical technique employed. The patients’ average BMI of 27.6 kg/m^2^ (range, 20.7-37.1 kg/m^2^, SD 4.0 kg/m^2^) in the menopaused group was significantly higher than the average BMI of 25.6 kg/m^2^ (range, 18.5-31.6 kg/m^2^, SD 3.0 kg/m^2^) in the non-menopaused group (P=0.005). Five patients (16.7%) in the menopaused group and 16 (20%) patients in the non-menopaused group were active smokers, with no significant difference (P=0.692). Three patients has diabetes (10%) in the non-menopaused group compared to 2 patients (2.5%) in the menopaused group (P=0.093). Four patients (13%) in the menopaused group and 4 patients (5%) in the non-menopaused group had hypertension (P= 0.13). Three non-menopaused patients were coagulopathic (3.75%), compared to none in the menopaused group which was statistically significant (P=0.019).

Breasts measurements were performed on a per-breast basis. The average preoperative SN-N distance of the right breast was 30.6 cm (SD 4.9 cm) in menopaused patients and 29.9 cm (SD 3.5 cm) in non-menopaused patients (P=0.576). The average left breast preoperative SN-N distance was of 30.1 cm (SD 4.5 cm) in menopaused patients and 29.9 cm (SD 3.4 cm) in non-menopaused patients (P=0.792). The average right breast preoperative NAC-IMF distance was 12.4 cm (SD 3.2 cm) in the menopaused group and 13.8 cm (SD 2.7 cm) in the non-menopaused group, whereas the average preoperative NAC-IMF distance was 12.6 cm (SD 3.4 cm) in menopaused patients and 14.0 cm (SD 2.6 cm) in non-menopaused patients (P=0.162). Generally, the menopausal breasts appeared to be slightly more ptotic, with a higher SN-N distance but shorter NAC-IMF distance. Additionally, the menopausal patients had smaller NAC diameters (Table 2).

**Table 2:** Preoperative measurements

	Non-menopaused	Menopaused	
	*n=156*	*n=60*	*P-value*
Sternal notch-to-nipple distance [cm], mean (SD)			
*Right breast*	29.9 (3.48)	30.64 (5.00)	0.576
*Left breast*	29.9 (3.38)	30.1 (4.54)	0.792
Inferior border of NAC to IMF distance [cm], mean (SD)			
*Right breast*	13.8 (2.73)	12.4 (3.17)	0.176
*Left breast*	14.1 (2.56)	12.6 (3.35)	0.162

### Intraoperative assessment measurements

Regarding the surgical technique, 7 patients (23.3%) in the menopaused group and 19 (23.8%) in the non-menopaused group underwent RM with superior pedicle (P> 0.99). Seventeen patients (56.7%) and 41 patients (51.3%) in the non-menopaused group underwent RM with superomedial pedicle (P=0.672). RM with a medial pedicle technique was employed in 1 patient in the menopaused group (3.3%) and 2 patients (2.5%) in the non-menopaused group (P>0.99). Five patients (16.7%) in the menopaused group and 18 patients (22.5%) in the non-menopaused group underwent RM with inferior pedicle (P=0.605). All operations were conducted following the Wise-pattern.

The average left breast resection weight was 595.2 g (SD 385.0 g, range 86-1970 g) in the menopaused group and 478.6 g (SD 243.1 g, range 50-1608 g) in the non-menopaused group, which was not statistically significant (P=0.142). The average right breast resection weights of 606.5 g (SD 395.7 g, range 72-1968 g) and 481.9 g (SD 254.9 g, range 48-1578 g) in menopaused and non-menopaused patients, respectively, were not significantly different (P=0.152).

The average operative time was 158.7 min (SD 39.0 min) for the menopaused group and 159 min (SD 200.0 min) for the non-menopaused group, with no differences across groups (P=0.973). All but 4 non-menopaused patients (5%) underwent bilateral RM (P=0.573) with no significant differences (Table 3).

**Table 3:** Operative characteristics

	Non-menopaused	Menopaused	
	*n=80*	*n=30*	*P-value*
**Operation side**			
*unilateral, n (%)*	4 (5%)	0 (0%)	0.573
*bilateral, n (%)*	76 (95%)	30 (100%)	
**Pedicles**			
*superior, n (%)*	19 (23.8%)	7 (23.3%)	1
*supero-medial, n (%)*	41 (51.3%)	17 (56.7%)	0.672
*medial, n (%)*	2 (2.5%)	1 (3.3%)	1
*inferior, n (%)*	18 (22.5%)	5 (16.7%)	0.605
**Operative time [min], mean (SD)**	159.0 (35.15)	158.7 (39.00)	0.973
**Resection weight [g], means (SD)**	480.2 (247.90)	600.8 (386.90)	0.044*
*Right breast*	481.9 (254.20)	606.5 (395.70)	0.152
*Left breast*	478.6 (243.10)	595.2 (385.00)	0.142

### Hospital stay and postoperative assessment

The hospital stay was significantly longer in the menopaused group, with an average hospital stay of 3.5 days (SD 1.8 days) compared to 2.6 days (SD 1.4 days) in the non-menopaused group (P=0.008).

The follow-up was similar across the groups, with an average of 10.2 months (SD 10.7 months) for menopaused patients, compared to 9.4 months (SD 8.1 months) for non-menopaused patients (P=0.890).

There was no statistically significant difference in the early complication rate of menopaused (10%, 3 patients) versus non-menopaused (7.5%, 6 patients) patients, (P=0.702). The rate of late complication rate did not differ across groups—with a rate of 53.3% (16 patients) in the menopaused group compared to 40% (32 patients) in the non-menopaused group (P=0.281).

One menopaused patient (3.3%) and in 3 non-menopaused patients (3.8%) had early complications that required surgical management (P>0.99). Surgical hematoma drainage was required for the menopaused and 2 non-menopaused patients. One non-menopaused patient returned to the operating room to address wound dehiscence.

Late complications that required surgical management occurred in 3 menopaused patients (10%) and 4 non-menopaused patients (5%), which was not significantly different (P=0.388). In the non-menopaused group, scar revision for scar hypertrophy was required in 3 non-menopaused patients, and 1 patient underwent scar revision owing to a depression that appeared under the scar. In the menopaused group, 2 patients underwent dog ears revision and 1 patient underwent surgical seroma evacuation.

The most common postoperative complication was scar hypertrophy, which was significantly more frequent in non-menopaused patients, occurring in 17 non-menopaused patients (21.3%) compared to 1 menopaused patient (3.3%; P=0.022). Four menopaused patients (13.3%) and 4 non-menopaused patients (5%) had postoperative breast asymmetry, with no differences across groups (P=0.210). Other common complications included hematoma and scar dehiscence, which occurred in 3 non-menopaused (3.8%) and 2 (6.7%) menopaused patients, and 4 non-menopaused (5.2%) and 2 (6.7%) menopaused patients, respectively, with no significant differences found across the groups (P=0.612 and P=0.672, respectively). Five patients (16.7%) in the menopaused group developed dog ears, which was significantly more frequent compared to 3 patients (3.75%) in the non-menopaused group (P=0.034). Additionally, in the menopaused group, 2 patients developed mastalgia (6.7%), 1 patient developed bruises and blisters (3.3%), and 1 patient had macromastia recurrence (3.3%). In the non-menopaused group, 1 patient developed mastalgia (1.3%), 2 patients had macromastia recurrence (2.5%), 1 coagulopathic patient developed pulmonary embolism (1.3%), 1 patient experienced NAC necrosis (1.3%), 2 patients reported areolar hypopigmentation and hyperpigmentation (1.3%), and 1 patient reported areolar hypersensibility (1.3%) (Table 4).

**Table 4:** Post-operative complications

	Non-menopaused	Menopaused	
	*n=80*	*n=30*	*P-value*
No complication, n (%)	42 (52.5%)	11 (36.7%)	0.198
≥1 complication, n (%)	38 (47.5%)	19 (63.3%)	
Early complications, n (%)	6 (7.5%)	3 (10%)	0.702
Late complications, n (%)	32 (40%)	16 (53.3%)	0.281
Hypertrophic scar, n (%)	17 (21.3%)	1 (3.3%)	0.022*
Breast asymmetry, n (%)	4 (5%)	4 (13.3%)	0.210
Hematoma, n (%)	3 (3.8%)	2 (6.7%)	0.612
Scar dehiscence, n (%)	4 (5.0%)	2 (6.7%)	0.672
Dog ear, n (%)	3 (3.8%)	5 (16.7%)	0.034*
Macromastia recurrence, n (%)	2 (2.5%)	1 (3.3%)	1
Postoperative pain, n (%)	1 (1.3%)	2 (6.7%)	0.180
NAC necrosis, n (%)	1 (1.3%)	0 (0%)	1
**Mean hospitalization time [days], SD**	2.6 (1.39)	3.5 (1.84)	0.008*

Using the median split separation for BMI and breast resection weight, we found no statistical difference in terms of complications. The BMI cutoff was set at 26 kg/m^2^. The complication rate was 58.2% in the subgroup with BMI ≥26 kg/m^2^ compared to 41.2% in the lower BMI subgroup, with no significant differences across the groups (P=0.127). The resection weight median split cutoff was set at 480 g, the subgroup with resection weight ≥480 g had complication rate of 58.2%, compared to 41.2% for resection weight <480 g, with no statistically significant differences across the subgroups (P=0.127). When performing a subgroup analysis of different factors, including diabetes, coagulopathy, and active smoker status, no significant associations were found with higher complication rates (Table 5).

**Table 5:** Complication rates and confounders

	Complications	
		*P-value*
BMI ≥26 kg/m^2^	58.2%	0.127
Control group	41.2%	
Resection weight ≥480 g	58.2%	0.127
Control group	42.6%	
Hypertension	88.9%	0.158
Control group	96.4%	
Diabetes	47.6%	0.057
Control group	54.4%	
Coagulopathy	96.4%	1
Control group	98.2%	
Active smokers	47.1%	1
Control group	49.1%	

**Figure 1:**
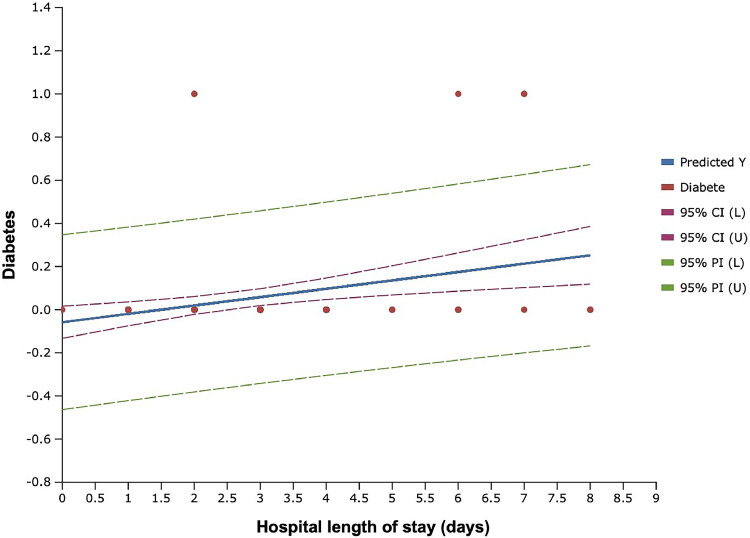
Scatter plot of hospital length of stay, according to presence of diabetes

**Figure 2:**
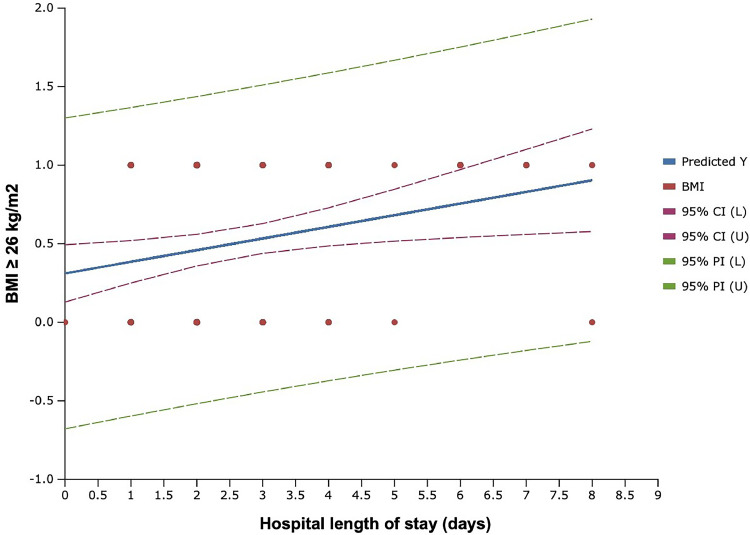
Scatter plot of hospital length of stay, according to BMI (kg/m^2^)

Regarding the correlation between comorbidities, operative characteristics and hospital stay, using regression analysis, we found that diabetes was associated with a significantly longer hospital stay (OR: 1.79, CI 95% 1.162 to 2.763, P=0.008). Similarly, BMI >26 kg/m^2^ was also associated with longer hospital stay (OR 1.40, CI 95% 1.061 to 1.857, P=0.018. We found no significant association between smoking status, hypertension, resection weight, and length of hospital stay (Table 6, Figure 1, Figure 2)

**Table 6:** Regression analysis between the length of hospital stay and patient characteristics, operative characteristics, and comorbidities

	Odds ratio	IC-95%	*P-value*
Diabetes	1.7920	1.162-2.763	0.008*
Hypertension	1.277	0.877-1.860	0.203
Active smokers	1.021	0.758-1.373	0.893
BMI >26 kg/m^2^	1.404	1.061-1.857	0.018*
Resection weight ≥480 g	1.234	0.947-1.608	1.234

## Discussion

Menopause, defined retrospectively as the cessation of menses for 12 months is not a point in time that appears abruptly and represents the cessation of ovarian production of oocytes. Rather, it is a dynamic process.^23^ Menopausal transition begins at the onset of irregular menstrual cycles and is typically characterized by the increase in follicular-stimulating hormone, as the hypothalamic-pituitary axis gradually loses sensitivity to positive and negative feedback by estrogen.^24^ Although individual experiences may vary, vasomotor symptoms, such as hot flushes, night sweats, and sleep disruption are recurring effects of the menopausal transition, the pace of which is influenced by chronological and ovarian aging. Despite not understanding the exact mechanisms leading to these symptoms, the drop in estrogen levels appears to be deeply correlated to them.^22^, ^23^, ^24^, ^25^ As longevity increases, it is expected that women will spend up to 40% of their lifetime in menopause, thus its non-vasomotor effects on the human body should not be neglected.^23^^,^^26^

The literature on the role of estrogen in wound healing and skin aging is vast, and numerous authors have noted that estrogen deficiency contributes to cutaneous aging, delayed wound repair, and impaired wound healing.^20^, ^21^, ^22^^,^^27^^,^^28^ Wound healing is segmented in 3 steps, namely hemostasis/inflammation, proliferation, and remodeling, and estrogen plays an important role in each of them. Whether it is by modifying the inflammatory response when affecting the neutrophil-macrophage cascade, by inducing the mobilization of endothelial progenitor cells from the bone marrow, accelerating re-epithelization, regulating the deposition of type I and III collagen, or by stimulating granular formation and proteolysis, an estrogen imbalance in any of these steps could result in irregular scaring.^20^, ^21^, ^22^ Furthermore, estrogen plays a crucial role by controlling oxidative stress, a factor which is known to be of critical importance in wound repair.^21^ Thus, menopause has been associated with poor or delayed wound healing and reduced vascularity in several studies.^20^^,^^22^

Postoperative complications in groups with contrasted estrogen level has been evaluated by some authors, such as Billon et al., who compared the postoperative outcomes and complications between patients under adjuvant anti-estrogen therapies and control patients. The authors found that patients taking tamoxifen and aromatase inhibitors had increased risk of developing wound healing complications with wound healing complication rate of 61% compared to 28% in patients who did not take the medications (P<0.001).^27^ Similarly, Lopez et al. reported that patients undergoing bilateral RM during the postovulatory phase of their menstrual cycle had an increased risk of irregular scarring and wound healing complications (P<0.005).^29^ Our findings correlate with the findings of these authors when considering hospital stay. Indeed, we found that the median hospital stay was significantly longer in menopaused patients compared to non-menopaused patients. Additionally, we found that diabetes and BMI ≥26 kg/m^2^ were significantly associated with longer hospital stay. As there were no significant differences across the menopaused and non-menopaused groups in terms of diabetes or BMI ≥26 kg/m^2^, this difference could be attributed to the menopausal status.

Moreover, we observed that postoperative dog ears were significantly more frequent in patients in the menopaused group (62.5%) compared to those in the non-menopaused group (37.5%). This could be explained by the loss of skin elasticity caused by the faltering levels of estrogen, as estrogen is associated with elastin production.^28^^,^^30^ Another factor which may affect breast elasticity is massive weight loss. In our study, 1 patient underwent bariatric surgery in the menopausal group, and no post-surgical complications were observed. Nevertheless, this patient was discharged 8 days postoperatively, which was longer than the average length of stay of 3.5 days. No patients underwent bariatric surgery in the non-menopausal group.

Although we observed a higher proportion of total complications in menopaused patients (63.3%) compared to non-menopaused patients (36.7%), the difference was not statistically significant. Instead, the tendency was reversed in one complication. Notably, hypertrophic scarring was significantly more frequent in non-menopaused patients (21.3%) compared to that in menopaused patients (3.3%). This finding suggests a deeper complexity regarding wound healing mechanisms. In contrast to the general hypothesis, the effect of estrogen on postoperative complications repartition might not necessarily and systematically be beneficial. Although estrogen could participate in the prevention of certain complications such as dog ears, other postoperative outcomes including hypertrophic scarring could appear more frequently among the non-menopaused patients.

Although not significant, we observed a higher complication rate in terms of postoperative breast asymmetry, scar dehiscence, mastalgia, hematoma, and gigantomastia recurrence among the menopaused patients versus non-menopaused patients. Although we may intuitively postulate that hormone replacement therapy (HRT) balances the risk of postoperative complications, in our study, 3 patients underwent HRT in the menopaused group. Moreover, late postoperative complications occurred in all 3 patients, notably scar dehiscence, scar hypertrophy, and breast asymmetry. Although this suggests that the benefits of HRT on postoperative complications may be limited, larger studies focusing on the effect of HRT on postoperative outcomes are warranted.

Some limitations of this study must be acknowledged. The retrospective design of this study constitutes a limitation. Furthermore, wound complication and healing may be associated with other factors, such as those inherent to the patient and those attributed to surgical technique. The addition of standard planned consultations could have provided us with more information regarding patient satisfaction over time. Finally, this study could have benefited from a larger menopaused group, as to optimize statistical significance. Age may, however, not be considered as a confounder, as menopause is an aspect of aging itself.

## Conclusion

To our knowledge, this is the first study evaluating the postoperative outcomes and complications of menopause after reduction mammoplasty. Interestingly, our finding nuances the current literature's description on the effect of estrogen on wound healing. It suggests that menopausal women are not at higher risk of postoperative complications compared to non-menopaused women, save for the dog ears. In fact, we have found that non-menopaused women experienced higher occurrence of hypertrophic scarring. Nonetheless, the menopausal status has been associated with longer hospitalization duration. A prospective study on this subject could be beneficial to better understand the effects of menopause on breast reduction surgery, long-term complications, and patient satisfaction.
